# Low-grade endometrial stromal sarcoma arising from extrauterine deep infiltrating endometriosis: a rare, but important differential diagnosis and short review of the literature

**DOI:** 10.1016/j.gore.2025.101985

**Published:** 2025-11-05

**Authors:** Lina Judit Schiestl, Simin Schadmand-Fischer, Evangelos Tagkalos, Udo Raab, Pia-Elisabeth Baqué, Nadja Hamscho, Friedrich Kommoss, Dennis Jung, Valerie Linz, Marcus Schmidt, Annette Hasenburg, Roxana Schwab

**Affiliations:** a Department of Obstetrics and Gynecology, University Medical Center of Johannes Gutenberg University Mainz, Germany; b Department of Radiology, University Medical Center of Johannes Gutenberg University, Mainz, Germany; c Departement of Nuclear Medicine, Universitiy Medical Center of Johannes Gutenberg University, Mainz, Germany; d MVZ Radiologie Nuklearmedizin am Bethanien-Krankenhaus, Frankfurt, Germany; e Institute of Pathology, Medizin Campus Bodensee, Friedrichshafen, Germany; f Department of General, Visceral and Transplant Surgery, University Medical Center of the Johannes Gutenberg University, Mainz, Germany; g Radiologie, Kreisklinik Groß Gerau, Germany

**Keywords:** Extrauterine LG-ESS, Endometriosis, Fertility-sparing surgery

## Abstract

**Background:**

Low-grade endometrial stroma sarcoma is a very rare mesenchymal neoplasm, that is usually found inside the uterine corpus. It is very uncommon that LG-ESS arises from extrauterine foci.

**Case presentation:**

We report the case of a 31-year-old patient diagnosed with extrauterine LG-ESS arising from deep infiltrating endometriosis. Primary symptoms, diagnostic workflow, operating method, and the outcome will be described, as well as a short review of the current literature about the optimal treatment for young LG-ESS patients within the reproductive window.

**Conclusions:**

Long-term surveillance is imperative for patients with LG-ESS given the potential for late recurrences. Although extrauterine LG-ESS represents an exceedingly rare gynecologic malignancy, establishing standardized treatment approaches is critical to optimize therapeutic efficacy and improve patient outcomes.

## Highlights

• LG-ESS is a rare uterine malignancy. Extrauterine LG-ESS arising from endometriosis is exceptionally uncommon.
• Casereport: a 32-year-old woman with extrauterine LG-ESS arising from endometriosis treated with maximum cytoreductive surgery and aromatase inhibitors.
• Clinical relevance: early recognition and correct diagnosis and treatment strategies are essential to improve outcomes.

## 1 Introduction

Low-grade endometrial stromal sarcoma (LG-ESS) is an extremely rare uterine malignancy, that belongs to the WHO classification of endometrial stromal neoplasms (Conklin and Longacre, 2014, Hoang et al., 2018, Lu and Chen, 2014). It accounts for less than 1 percent of all uterine malignant findings (Chan et al., 2008). The most important prognostic factors are tumor stage and the patient’s age (Abu-Rustum et al., 2021, Chew and Oliva, 2010). At diagnosis, approximately 65 % of patients are in FIGO stages I–II, while about 35 % are in stages III–IV (Chan et al., 2008). For individuals diagnosed with tumor stages I–II, the five-year survival rate exceeds 90 %. However, for those in stages III–IV, the survival rate drops to around 50 %. The median time until recurrence occurs ranges from 5.4 to 9.3 years for stages I and II, but drops to just 9 months for stages III and IV, according to FIGO classifications prior to 2009 (Amant et al., 2003, Kim et al., 2008). Clinical presenting symptoms are most likely: abnormal uterine bleeding in pre- and postmenopausal patients and abdominal pain and distension (Liao et al., 2001, Nordal and Thoresen, 1997, Schwartz et al., 1985).

Despite advances in modern imaging techniques, distinguishing between benign and malignant uterine lesions remains challenging (Amant et al., 2009, Kitajima et al., 2010, Rha et al., 2003). Imaging features of uterine sarcomas are largely nonspecific. This also applies to low-grade endometrial stromal sarcomas, for which ultrasound, computed tomography (CT), and magnetic resonance imaging (MRI) typically fail to demonstrate distinctive diagnostic features (Amant et al., 2009). In ultrasound heterogenic hypoechoic masses with endometrial involvement can often be seen, as well as solid masses with cystic degeneration (Gadducci et al., 2023, Gandolfo et al., 2000). In general, in MRI with diffuse weighted imaging, the only characteristic pattern for uterine sarcomas are worm-like projections in the vessels or along ligaments (Gadducci et al., 2023, Tamai et al., 2008). Only a few case series have examined the characteristic imaging findings of uterine sarcomas using fluorodeoxyglucose positron emission tomography (^18^F-FDG PET). Zhao et al. (2013) reported that combined ^18^F-FES and ^18^F-FDG PET imaging may assist in differentiating benign from malignant mesenchymal uterine tumors, as malignant lesions generally exhibit increased glucose metabolism and reduced estrogen receptor expression. Nonetheless, data specifically addressing low-grade endometrial stromal sarcoma are scarce, and PET findings in this subgroup remain nonspecific (Zhao et al., 2013). The recommended work-up by ESGO/ EURACAN/GCIG for smooth muscle tumors includes transvaginal or transabdominal ultrasound performed by an experienced examiner, or contrast-enhanced pelvic MRI. In cases where oncological surgery is indicated, a CT scan is recommended both preoperatively and postoperatively (Ray-Coquard et al., 2024).

Histopathological LG-ESS cells resemble endometrial stroma during in its proliferate phase, but tend to show invasive growth and lymphovascular invasion (Abu-Rustum et al., 2021, Hoang et al., 2018). According to the ESGO guidelines for the management of patients with uterine sarcomas, the presence of more than three permeative infiltrations of the myometrium and/or evidence of lymphovascular invasion should be discovered for diagnosis (Ray-Coquard et al., 2024). The malignant cells show positivity for CD10, WT1, estrogen and progesterone receptor (Abu-Rustum et al., 2021, Denschlag et al., 2015). Most likely LG-ESS are found in the uterine corpus (Micci et al., 2021). LG-ESS of extrauterine origin, e.g. the pelvis, is a very rare phenomenon (Leath et al., 2007, Wu et al., 2022). The most common explanation for the origin of extrauterine LG-ESS, is that the tumorous cells originate from extrauterine endometriosis (Jung et al., 2009, Wu et al., 2022), as endometriosis was found in approximately 50 % of extrauterine LG-ESS (Köhler and Evert, 2009). Endometriosis on the other hand is a quite common benign disorder occurring in more than 10 % of all fertile women (Shafrir et al., 2018, Soliman et al., 2018). Malignant findings arising from endometriosis are very uncommon and only occur in 0.7–0.1 % of all investigated cases (Baiocchi et al., 1990). Within this very small number of cases of malignancy arising from endometriosis, LG-ESS is the least common. No more than 80 cases of ESS arising in endometriosis have been reported in the English literature (Lan et al., 2012, Wu et al., 2022).

## 2 Case

We present a 32-year-old woman with LG-ESS arising from deep-infiltrating endometriosis (DIE). Initial symptoms, imaging modalities, histological findings, disease progression, operation technique and the outcome after final surgery are reported.

## 3 Casepresentation

### 3.1 Subsection

**Fig. 1:**
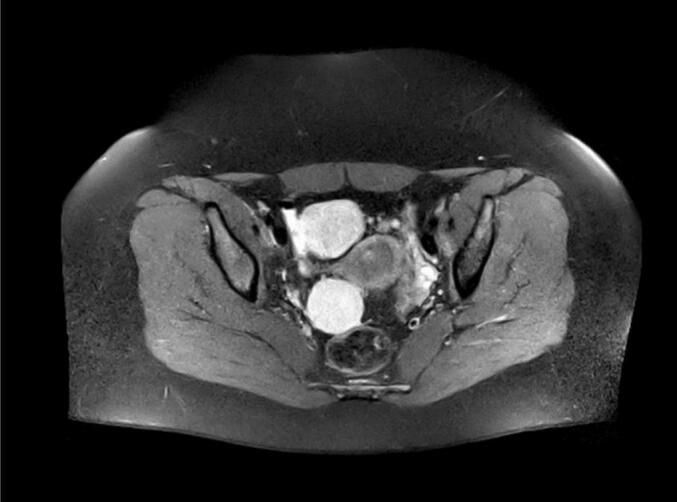
MRI before laparoscopy.

**Fig. 2:**
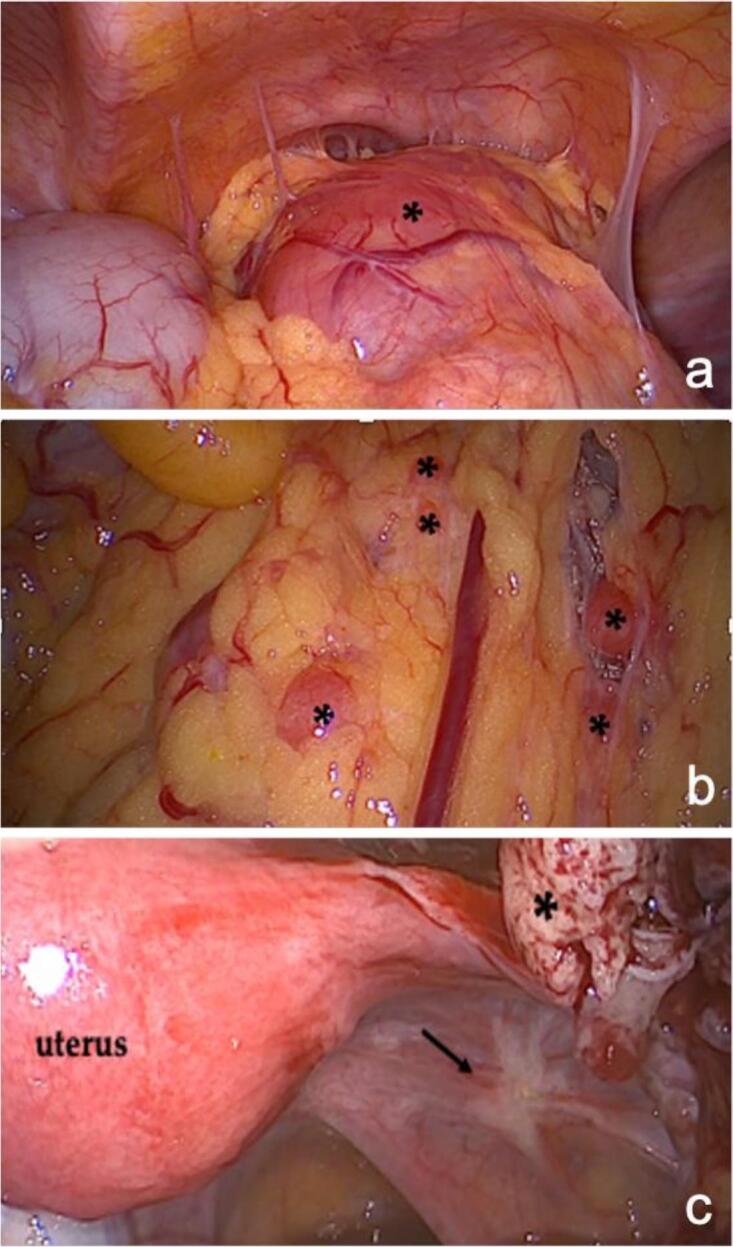
Laparoscopy. A) A large tumour mass in the omentum adjacent to the peritoneum of the urinary bladder. B) Small disseminated tumour masses within the omentum. C) Necrotic tumour mass adjacent to the uterine wall (*) and deep infiltrating endometriosis (DIE) on the right lateral pelvic wall (black arrow).

**Fig. 3:**
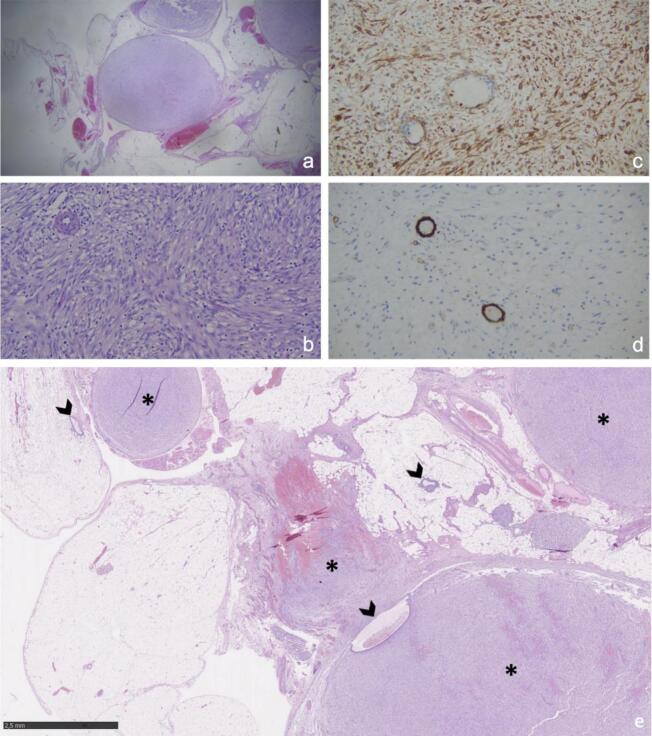
Pathology a) Peritoneal manifestation of a low-grade endometrioid stromal sarcoma: nodular spindel cell proliferates surrounded by mature adipose tissue (HE, x25). b) Spindle cell proliferate of low-grade endometrial stromal sarcoma: low degree of pleomorphy, characteristic arteriolar vascular pattern (HE, x200). c) Spindle cell proliferate of low-grade endometrial stromal sarcoma, immunohistochemically diffusely positive for CD10 (stromal marker). d) Spindle cell proliferate of low-grade endometrial stromal sarcoma, immunohistochemically negative for caldesmon (smooth muscle marker, vessel walls as internal positive control positive). e) direct association between an endometriotic focus and the stromal sarcoma (arrow: endometriotic foci in the omentum, star: LG-ESS).

Initially, the patient complained about unexplained infertility and gastrointestinal problems. An additional ultrasound examination revealed two indolent hypoechoic cystic formations in the pelvis. In a follow-up CT scan two rather uncharacteristic solid masses were detected. An additionally performed MRI (Fig. 1) showed multiple T2 hyperintensities with diffusion disturbance in the close-by peritoneum. In the consecutively performed diagnostic laparoscopy, multiple hypervascularized lesions in the lower and upper abdominal cavity were found (Fig. 2). Because of intraoperative suspicion of malignancy, only a biopsy was performed, and resection was deferred. Histopathologic examination of the biopsy revealed a uniform proliferation of small, round to ovoid cells with scant cytoplasm and bland nuclei, resembling proliferative-phase endometrial stroma. Mitotic activity was low, and necrosis was absent. The tumor showed an infiltrative, tongue-like growth pattern with permeation into adjacent soft tissue and delicate, spiral arteriole–type vasculature. Residual endometrial-type glands and stroma contiguous with the neoplasm were identified, supporting origin from deep infiltrating endometriosis rather than uterine extension. Immunohistochemical analysis of the biopsy specimen demonstrated strong nuclear expression of WT1, estrogen receptor (ER; IRS 12), and progesterone receptor (PR; IRS 12), with focal positivity for CD10 and CD34 within the stromal component. The tumor cells were negative for desmin, smooth muscle actin (SMA), h-caldesmon, S100, and CD31. Molecular pathological analysis revealed no mutations in codons 28–46 of the CTNNB1 (β-catenin) gene. These findings supported the diagnosis of LG-ESS arising from DIE. A follow-up MRI after three months (Fig. 3) showed clear signs of tumor progression. To obtain more information a PET/CT was performed (Fig. 5). Based on the previous diagnostic measures described above the interdisciplinary tumor conference recommended maximum cytoreductive surgery. Surgery was performed with oncologic intent, consisting of maximal cytoreductive procedures without fertility preservation (total hysterectomy with bilateral salpingo-oophorectomy, omentectomy, peritoneal biopsy, and excision of additional suspicious lesions), achieving a macroscopically complete (R0) resection of both the tumor and the deep infiltrating endometriosis. Extensive histopathological sampling of the hysterectomy specimen revealed no evidence of low-grade endometrial stromal sarcoma (LG-ESS) arising from the uterine corpus. The absence of tumor infiltration in the uterine wall, despite metastatic lesions in the uterine serosa, supports the conclusion that the LG-ESS originated from extrauterine endometriotic foci. The final tumor stage was FIGO IIIB. Adjuvant endocrine therapy with aromatase inhibitors was initiated. Six, 12 and 18 months after diagnosis, a follow-up MRI of the pelvis and CT-scans of the thorax showed no local or distant tumor recurrence.

### 3.2 Images

**Fig. 4:**
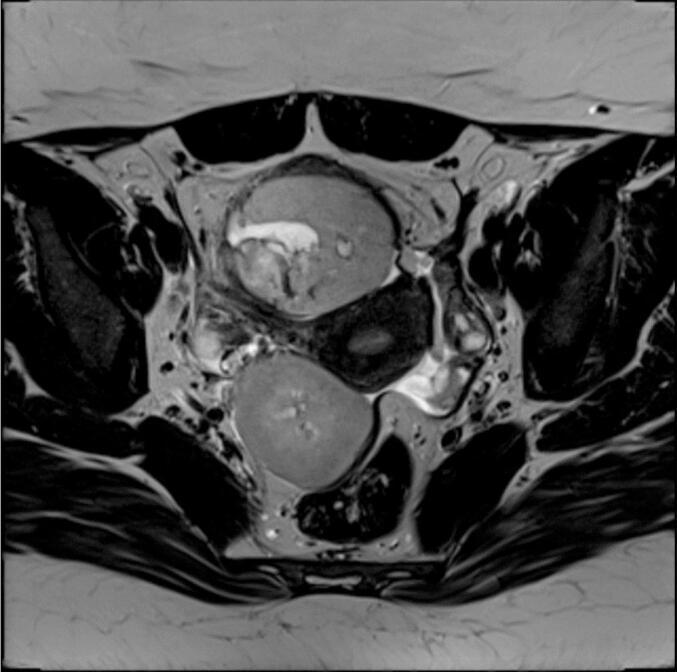
Preoperative MRI.

**Fig. 5:**
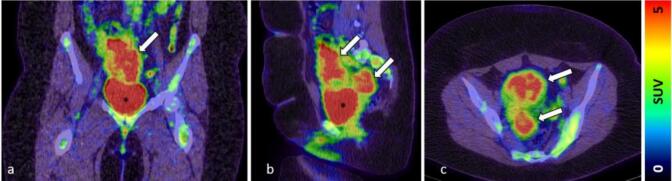
PET/CT (206 MBq ^18^F-FDG) a coronal fusion, b sagittal fusion, c transaxial fusion; *:bladder; white arrows: Omental tumor lesions with increased glucose metabolism compatible with malignancy (SUV: standardized uptake value).

## 4 Discussion

Low-grade endometrial stromal sarcoma (LG-ESS) is an extremely rare malignant neoplasm, and extrauterine LG-ESS is even less common. Due to the limited data available, there is currently no consensus on a standardized diagnostic work-up or treatment approach. According to NCCN and German guidelines, and as demonstrated in the present case, the diagnosis of LG-ESS is confirmed through histopathological evaluation showing characteristic spindle-cell proliferation with myometrial and/or lymphovascular invasion, supported by immunohistochemical positivity for CD10, estrogen receptor (ER), and progesterone receptor (PR) (Abu-Rustum et al., 2021, Denschlag et al., 2022).

While most LG-ESSs originate within the uterus, emerging evidence indicates that malignant transformation of endometriosis—though uncommon—may give rise to a range of neoplasms, including endometrioid carcinoma, clear cell carcinoma, and, rarely, endometrial stromal sarcoma. The proposed mechanism involves neoplastic transformation of ectopic endometrial stromal cells within endometriotic foci under chronic hormonal and inflammatory stimulation (Ioannidou et al., 2025, Mccluggage, 2020). In the present case, the identification of residual endometrial-type glands and stroma contiguous with the neoplastic proliferation, in the absence of a uterine primary, supports the interpretation of an LG-ESS arising from DIE rather than representing metastatic spread or direct uterine extension. These findings align with Sampson and Scott’s classical criteria for malignant transformation in endometriosis, emphasizing histologic continuity between benign and malignant endometrial elements and exclusion of an alternative primary site (Gadducci and Zannoni, 2020).

Accurate and timely diagnosis of LG-ESS remains challenging. The standard treatment consists of total hysterectomy with bilateral salpingo-oophorectomy, which offers optimal oncologic control (Denschlag et al., 2022, Thiel and Halmen, 2018). Fertility-sparing surgery for uterine sarcomas confined to the uterus is considered experimental and is supported only by small retrospective studies and isolated case reports. Consequently, conservative management is not routinely recommended, and women desiring fertility preservation should receive comprehensive counseling regarding the limited evidence, potential oncologic risks, and high likelihood of recurrence (Dondi et al., 2021). Nevertheless, several small case series suggest that fertility preservation may be feasible in carefully selected patients with early-stage, intrauterine LG-ESS (Bai et al., 2014, Gu et al., 2021, Laurelli et al., 2015, Noventa et al., 2015). In contrast, numerous other reports document high recurrence rates following conservative surgery, often necessitating subsequent radical intervention. A 2021 systematic review reported a 54 % recurrence rate among women with LG-ESS who underwent fertility-sparing surgery, based on pooled data from 210 cases of uterine sarcomas managed conservatively (Dondi et al., 2021). Although most patients were alive at the end of follow-up, these outcomes require cautious interpretation due to the relatively short median follow-up of less than five years (Xie et al., 2017, Zheng et al., 2020). Importantly, delayed fatal relapses have been described years after initial fertility-sparing treatment (Koskas et al., 2009, Morimoto et al., 2015). By definition, extrauterine low-grade endometrial stromal sarcoma is classified as at least FIGO stage II, reflecting a more advanced stage at the time of diagnosis when compared with uterine low-grade endometrial stromal sarcoma, of which approximately 65 % of cases remain confined to the uterus at presentation. Additionally, the stage of the disease is recognized as the most important prognostic factor for survival (Chan et al., 2008). Consequently, fertility-sparing surgery, which might significantly impact survival, should not be recommended for patients with extrauterine LG-ESS. Instead, a maximal surgical effort aimed at achieving complete resection or maximal cytoreduction is advised for cases with extrauterine manifestations (Gadducci et al., 2008, Gadducci et al., 1996).

In the current case, the presence of visible metastatic foci (Fig. 3, Fig. 4, Fig. 5) precluded the possibility of fertility-sparing surgery. There is substantial international literature indicating that total hysterectomy, with or without bilateral salpingo-oophorectomy (BSO), represents the safest approach for all patients (Amant et al., 2007). Lymph node metastases occur in up to 12.6 % of women with low-grade endometrial stromal sarcoma (LG-ESS), yet the presence of lymph node metastases has not been shown to influence overall survival (Chan et al., 2008, Shah et al., 2008, Wu et al., 2020). While lymphonodectomy for enlarged or suspect lymph nodes is recommended, there is no evidence supporting the routine performance of systematic lymphonodecto-my (Chan et al., 2008, Denschlag et al., 2022). Furthermore, a study involving 10 patients with extrauterine LG-ESS did not observe any survival benefit from lymphadenectomy (Wu et al., 2022). Adjuvant treatment with aromatase inhibitors, such as letrozole, is recommended due to the high expression of estrogen and progesterone receptors in LG-ESS. Performing bilateral salpingo-oophorectomy (BSO) may obviate the need for concurrent treatment with GnRH analogs during aromatase inhibitor therapy in premenopausal women (Denschlag et al., 2022, Reich et al., 2007, Wu et al., 2022).

Additionally, adjuvant endocrine treatment has been shown to reduce the risk of recurrence in women with extrauterine LG-ESS.

Adjuvant treatment options for women with low-grade endometrial stromal sarcoma (LG-ESS) are limited, and the evidence supporting their efficacy remains inconclusive. Current data indicate that radiotherapy and chemotherapy provide no clear survival benefit and may even negatively affect long-term outcomes (Bai et al., 2014, Barney et al., 2009, Reed et al., 2008, Seagle et al., 2017, Wu et al., 2020, Wu et al., 2022, Zhou et al., 2015), although radiotherapy can improve local disease control in selected cases (Mahdavi et al., 2009, Malouf et al., 2010, Sampath et al., 2010). Given the high expression of estrogen and progesterone receptors in LG-ESS, adjuvant endocrine therapy with aromatase inhibitors such as letrozole is often recommended (Denschlag et al., 2022, Reich et al., 2007, Wu et al., 2022). In premenopausal women, bilateral salpingo-oophorectomy may obviate the need for concurrent gonadotropin-releasing hormone (GnRH) analogs during aromatase inhibitor therapy. Furthermore, hormonal adjuvant therapy has been associated with a reduced risk of recurrence, particularly in women with extrauterine LG- ESS (Wu et al., 2022).

In the present case, the patient exhibited no signs of recurrence after a follow-up period of 20 months. Thus, adjuvant therapy with letrozole following maximal surgical cytoreduction—including total hysterectomy with bilateral salpingo-oophorectomy and excision of all macroscopic tumor and endometriotic lesions, achieving an intraoperative complete (R0) resection—proved to be an effective treatment approach in this rare subset of women with low-grade endometrial stromal sarcoma arising from deep infiltrating endometriosis.

## 5 Conclusions

LG-ESS arising in endometriotic lesions is, by definition, extrauterine. Due to the limited evidence available, management strategies are generally extrapolated from those applied to extrauterine LG-ESS. As these tumors often present at an advanced stage, maximal cytoreductive surgery—including hysterectomy, bilateral salpingo-oophorectomy, and excision of all tumor and endometriotic foci—followed by adjuvant endocrine therapy with aromatase inhibitors, is recommended. This approach should be thoroughly discussed with patients. Long-term follow-up is essential in women with LG-ESS due to the possibility of late recurrences. Although extrauterine LG-ESS is a very rare gynecological malignancy, it is crucial to standardize and thereby optimize treatment options to improve patient outcomes.

## 6 Institutional review board statement

Not applicable.

## 7 Informed consent statement

Informed consent was obtained from all subjects involved in the study.

## CRediT authorship contribution statement

**Lina Judit Schiestl:** Writing – original draft, Visualization, Project administration, Investigation, Formal analysis, Data curation, Conceptualization. **Simin Schadmand-Fischer:** Visualization. **Evangelos Tagkalos:** Visualization, Conceptualization. **Udo Raab:** Resources. **Pia-Elisabeth Baqué:** Visualization, Resources. **Nadja Hamscho:** Resources. **Friedrich Kommoss:** Resources. **Dennis Jung:** Methodology, Conceptualization. **Valerie Linz:** Writing – review & editing. **Marcus Schmidt:** Writing – review & editing, Resources. **Annette Hasenburg:** Writing – review & editing, Validation, Supervision. **Roxana Schwab:** Writing – review & editing, Supervision, Resources, Methodology, Investigation, Data curation, Conceptualization.

## Funding

This research received no external funding.

## Declaration of competing interest

The authors declare that they have no known competing financial interests or personal relationships that could have appeared to influence the work reported in this paper.
